# Ocular Inflammation in Uveal Tract in Aged Obese Type 2 Diabetic Rats (Spontaneously Diabetic Torii Fatty Rats)

**DOI:** 10.1155/2014/629016

**Published:** 2014-09-14

**Authors:** Yusuke Kemmochi, Katsuhiro Miyajima, Takeshi Ohta, Tomohiko Sasase, Yuzo Yasui, Kaoru Toyoda, Kochi Kakimoto, Toshiyuki Shoda, Akihiro Kakehashi

**Affiliations:** ^1^Japan Tobacco Inc., Central Pharmaceutical Research Institute, Toxicology Research Laboratories, 23 Naganuki, Hadano, Kanagawa 257-0024, Japan; ^2^Japan Tobacco Inc., Central Pharmaceutical Research Institute, Biological/Pharmacological Research Laboratories, Osaka 569-1125, Japan; ^3^Department of Ophthalmology, Saitama Medical Center, Jichi Medical University, Saitama 330-8503, Japan

## Abstract

We report uveitis observed in an obese type 2 diabetes rat model, Spontaneously Diabetic Torii Lepr^fa^ (SDT fatty) rats aged over 50 weeks. The eyes of SDT fatty rats (16 animals: 7 males and 9 females with 50 or 60 weeks of age) were examined histopathologically. Infiltration of inflammatory cells in the uveal tract was observed in 13 of 16 animals. One female showed severe inflammation affecting the entire uveal tract including the iris, ciliary body, and choroid with a variety of inflammatory cells (neutrophils, lymphocytes, and macrophages). Those changes clinically mimic the findings of diabetic iridocyclitis in diabetic patients. Uveitis associated with diabetes can occur in diabetic patients but the pathogenesis still remains unknown. Since increased extramedullary hematopoiesis in the spleen and abscess in the genital and lower urinary tracts were observed in some SDT fatty rats, increased susceptibility to infection, prolongation of inflammatory states, and disorders of the immune system were considered to be possible factors of the uveitis in aged SDT fatty rats. There have been few reports on how diabetes has influence on the development of uveitis associated with bacterial infection. The SDT fatty rat can be an animal model to investigate diabetes-associated uveitis.

## 1. Introduction

Uveitis can be defined as inflammation in the uveal tract in the eye. Uveitis is the major cause of severe visual impairment in human and has been estimated to account for 5% to 15% of all cases of total blindness in the United States [1]. Diabetes has long been known to increase the chances of a variety of eye diseases, including retinopathy, cataracts, glaucoma, and uveitis [2]. However, the mechanism of cause of diabetes-associated uveitis still remains unknown.

The Spontaneously Diabetic Torii (SDT) fatty rat, established by introducing the fa allele of the Zucker fatty rat into the SDT rat genome, is a new model of obese type 2 diabetes [3]. The SDT-fa/fa (SDT fatty) rat shows overt obesity, hyperglycemia, and hyperlipidemia at a young age as compared with the other diabetes rat models [4, 5]. With an early incidence of diabetes mellitus, diabetes-associated complications in SDT fatty rats were observed at younger ages compared with those in the SDT rats [4–7]. Spontaneous ocular lesions of the SDT fatty rats up to 40 weeks of age have been reported but not in aged SDT fatty rats over 50 weeks of age [4–6]. We examined the eye of the SDT fatty rat over 50 weeks of age histopathologically in both sexes and report its characteristics.

## 2. Materials and Methods

Male and female SDT-fa/fa (fatty) rats and age-matched SDT-+/+ (SDT) rats were used from our colonies and age-matched Sprague-Dawley (SD) rats were purchased from CLEA Japan, Inc. (Tokyo, Japan). The rats were housed in a climate-controlled room with a temperature of 23 ± 3°C, humidity of 55 ± 15%, and a 12 h lighting cycle. A basal diet (CRF-1, Oriental Yeast Co., Ltd., Tokyo, Japan) and water were provided* ad libitum*. All the experiments received prior approval from the committee for the human care and use of animals of our laboratory, in accordance with the Standards Relating to the Care and Management of Experimental Animals. Necropsy was performed on the SDT fatty rats, SDT rats, and SD rat at 50 and 60 weeks of age. All animals were sacrificed by exsanguinations under light ether anesthesia. The eyes were sampled and fixed in formalin-glutaraldehyde mix fixative, then embedded in paraffin, sectioned, stained with hematoxylin and eosin (HE), and examined histopathologically (SDT fatty rats; 16 (7 males and 9 females), SDT rats; 17 (6 males and 11 females), and SD rat; 19 (9 males and 10 females)).

## 3. Results and Discussion

At necropsy, severe opacity of the lens was observed in all of the SDT fatty rats in both sexes (data not shown). A large-sized spleen and abscess surrounded in the genital and lower urinary tracts were also observed in SDT fatty rats in both sexes. Some SDT fatty rats also showed swelling and thickening of the foot pad, the so-called “bumble foot,” possibly due to the increase in body weight and chronic stimulus from the wire-bottomed cages on the foot pad.

**Figure 1:**
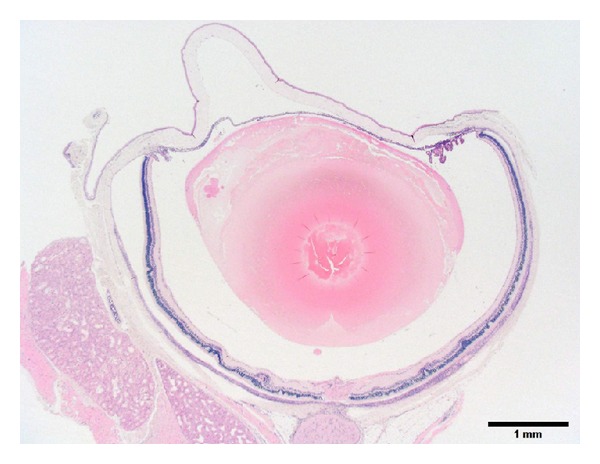
Photomicrograph of the eye of a 50-week-old female SDT fatty rat. Low power magnification of an eye showing deformation of lens, disarrangement of the retina (retinal fold), and infiltration of inflammatory cells in the uveal tract. Bar = 1 mm. Hematoxylin and eosin.

**Figure 2:**
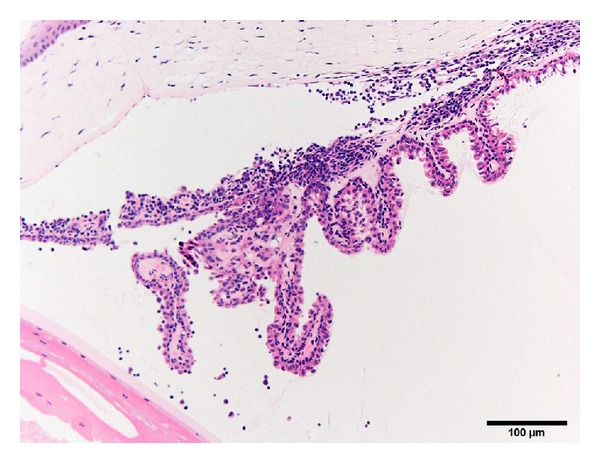
Photomicrograph of the ciliary body/iris of the eye from a 50-week-old female SDT fatty rat. There is infiltration of inflammatory cells in the ciliary body, iris, the anterior and posterior chambers, and the angle of anterior chamber. Bar = 100 *μ*m. Hematoxylin and eosin.

**Figure 3:**
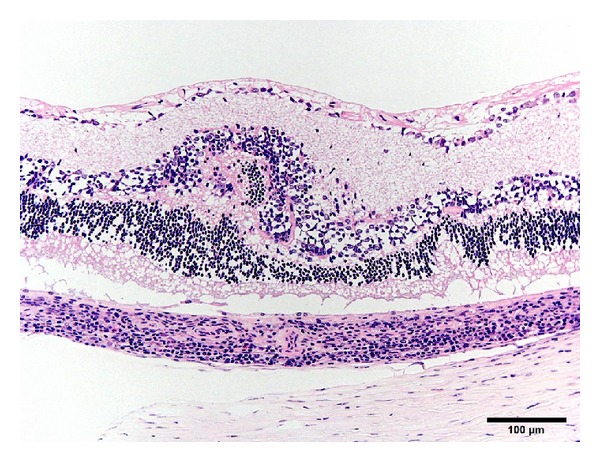
Photomicrograph of the retina and choroid of the eye from a 50-week-old female SDT fatty rat. There is infiltration of inflammatory cells in the choroid and the retinal fold characterized by disarrangement and thickness of the retinal layers. Bar = 100 *μ*m. Hematoxylin and eosin.

**Figure 4:**
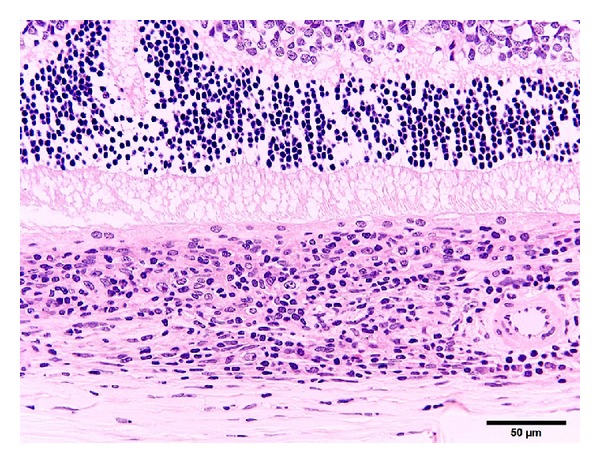
Photomicrograph of the choroid of the eye from a 50-week-old female SDT fatty rat. There is diffuse infiltration of inflammatory cells in the choroid. Inflammatory cells are partly spreading to the surrounding area in the sclera. Bar = 50 *μ*m. Hematoxylin and eosin.

**Figure 5:**
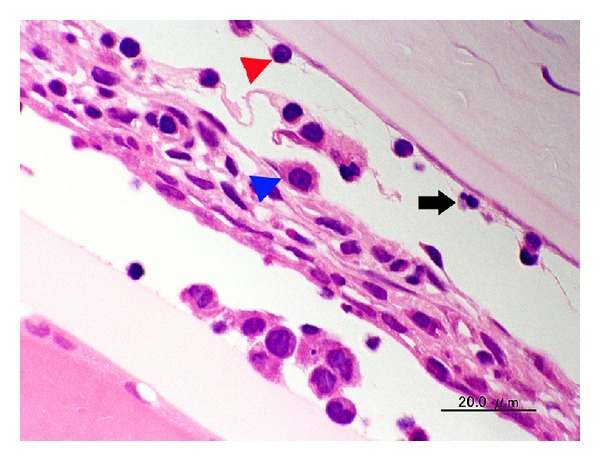
Photomicrograph of the iris of the eye from a 50-week-old female SDT fatty rat. There are inflammatory cells including a neutrophil (arrow), a macrophage (blue arrow head), and a lymphocyte (red arrow head) in the iris and the anterior and posterior chambers. Bar = 20 *μ*m. Hematoxylin and eosin.

Histologically uveitis, including infiltration of inflammatory cells in the uveal tract in the eye, was observed in 13 of 16 SDT fatty rats of both sexes (Table 1). The inflammatory cells consisted of neutrophils, macrophages, and lymphocytes. The finding was observed mainly in the choroid, but one female showed severe inflammation throughout the iris and ciliary body (Figures 1, 2, 3, 4, and 5). The changes clinically mimic the findings in diabetic iridocyclitis in diabetic patients. Advanced cases with these findings result in posterior synechia and following this are iris bombe and acute glaucoma. In the SDT rats, very slight inflammatory cell infiltration was also observed in the iris and ciliary body but no findings were observed in the choroid. There were increases in the incidence and degree of thickened and disarranged retinal layers (retinal fold) and degenerative lens fiber (cataract) in the SDT fatty rats, compared with those in the SDT rats. The enlarged spleen in the SDT fatty rats was histologically characterized by severely increased extramedullary hematopoiesis (EMH). Myeloid and granulocyte components were predominant in the EMH in the spleen of the SDT fatty rats. A lesion in the foot pad showed histologically ulcer and acanthosis of the epidermis with inflammation (ulcerative pododermatitis).

Uveitis is an inflammatory ocular disease of the uveal tract which is composed of the iris, choroid, and ciliary body. Uveitis can be caused by various factors including infectious or noninfectious (autoimmune) processes, often associated with systemic disease [8]. Although retinopathy is a major diabetic complication in the eye, uveitis can also be observed in diabetic patients [8, 9]. Diabetic patients, 1% to 6%, had uveitis in the iris, the most common localization of uveitis in diabetic humans [8–10]. Destruction of the blood retina barrier, increased blood permeability, and increased susceptibility to infection are considered to be involved in diabetes-associated uveitis but the details of pathogenesis still remain unknown [11–13].

Diabetes has been identified as an important risk factor for infection [14]. Hyperglycemia can induce poor wound healing and increased susceptibility to infection [15]. Once infection occurs in diabetic conditions, inflammation tends to last because diabetes prolongs the inflammatory response to a bacterial stimulus through cytokine dysregulation [16]. Endotoxin induced uveitis (EIU) is known as an experimental animal model of uveitis initiated by injection of lipopolysaccharide (LPS) [17, 18]. Although inflammatory response in this model lasts only for 72 hours, EIU rats with diabetes induced by streptozotocin (STZ) tended to have a prolonged inflammatory response [16].

EMH is commonly seen in the normal spleen, especially in young rodents as compared to aged rodents [19, 20]. EMH consists mainly of erythroid and myeloid precursors, indicating physiopathological responses secondary to hemorrhagic and inflammatory conditions, respectively. Increased EMH in the spleen in aged SDT fatty rats could have resulted from infections elsewhere in the body because some SDT fatty rats had severe inflammation in the foot pad and in the lower urinary tract, which could have been caused by bacterial infection. Another hematologic analysis on young SDT fatty rats showed that the leukocyte count (WBC) in SDT fatty rats was significantly higher than that in SD rats [6]. Decreased resistance to infection and a dysregulated inflammatory response may be responsible for the systemic condition of the SDT fatty rats.

Type 2 diabetes has been redefined as an inflammatory disease. The state of the immune system can be altered in obesity and type 2 diabetes, with apparent changes occurring in adipose tissue, liver, pancreatic islets, vasculature, and circulating leukocytes [21]. Type 2 diabetes is associated with higher serum levels of inflammatory cytokines (tumor necrosis factor, TNF) [22–24]. This may be due to the production of TNF in adipose tissue [25], the activity of advanced glycation end products, or enhanced cytokine production caused by the indirect effects of hyperinsulinemia or hyperglycemia [26, 27]. It is reported that TNF mRNA levels in SDT fatty rats tended to be increased as compared with those in SDT rats [28].

Aldose reductase inhibitors, which are a class of drugs to prevent eye and nerve damage in diabetic patients, are associated with a decrease in ocular inflammatory complications such as uveitis [29]. When metformin was given to the experimental uveitis model rats induced by an endotoxin, which mimicked the inflammatory effects of bacterial infection, endotoxin induced uveitis was inhibited or was prevented in the metformin group [30]. These cases indicate that diabetes might be one of the contributing factors of uveitis because drugs for diabetes not only improved disorders of carbohydrate metabolism but also decreased ocular inflammation.

Hyperglycemia and dyslipidemia arise at a younger age in the SDT fatty rats compared with other diabetic rat models. Severe and prolonged metabolic disorders in the SDT fatty rats may result in uveitis possibly due to bacterial infection and dysregulated immune system. There are not many reports investigating the association between infection and uveitis by using diabetic animal models, possibly because the mechanism of uveitis is considered to be complicated and multifactorial. The SDT fatty rat is a possibly useful model for investigating the mechanisms of uveitis associated with obesity and diabetes mellitus, especially related to bacterial infection. Whether the SDT fatty rats have stronger susceptibility to infection followed by systemic inflammation leading to uveitis compared to other diabetic models still remains unknown. Further investigation to elucidate potential relationship between metabolic disorder and uveitis in the SDT fatty rats is required.

## Figures and Tables

**Table 1:** Histopathological findings of the eyes in SDT fatty rats at weeks 50 and 60 of age.

Organ Pathological finding	Sex	Male															Female														
	Strain	SD					SDT					SDT fatty					SD					SDT					SDT fatty				
	Number of animals	9					6					7					10					11					9				
	Grade	−	±	+	2+	3+	−	±	+	2+	3+	−	±	+	2+	3+	−	±	+	2+	3+	−	±	+	2+	3+	−	±	+	2+	3+
Choroid																															
Infiltration, inflammatory cell		9					6					1	1	3	2		10					11					2	1	5		1
Iris/ciliary body																															
Infiltration, inflammatory cell		9					4	2				5	2				10					6	5					3	5	1	
Deposit, hemosiderin, and trabecular meshwork		9					3	1	2			4	2	1			10					6	5				8	1			
Retina																															
Retinal fold		8	1				3	1	2					4	3		10					9	1	1				1	3	5	
Lens																															
Deformation		9							5	1					7		10					4	2	5			1		2	6	
Degeneration, lens fiber (vacuolation/morgani body)		9								6						7	10					3	4	4			1		2	5	1
Hypertrophy/proliferation, epithelium		9					1	2	3			3		3	1		10					11					7		2		

−: negative, ±: very slight, +: slight, 2+: moderate, and 3+: severe.
